# Supplementary material for: Chemical Adsorption Strategy for DMC-MeOH Mixture Separation
Source: Molecules. 2021 Mar 19;26(6):1735. doi: 10.3390/molecules26061735 (PMC8003759; doi:10.3390/molecules26061735)

molecules

Supplementary Material

Chemical Adsorption Strategy for DMC-MeOH Mixture Separation†

Fucan Zhang [1,2], Ping Liu [1], Kan Zhang [1] and Qing-Wen Song [1,*]

[1] State Key Laboratory of Coal Conversion, Institute of Coal Chemistry, Chinese Academy of Sciences, Taiyuan 030001, China; 17865422752@163.com (F.Z.); pingliu@sxicc.ac.cn (P.L.); zhangkan@sxicc.ac.cn (K.Z.)
[2] State Key Laboratory of Heavy Oil Processing, College of Chemical Engineering and Environment, Chinese China University of Petroleum (Beijing), Beijing 102249, China
* Correspondence: songqingwen@sxicc.ac.cn
† Dedicated to the 100th anniversary of Chemistry at Nankai University

Table of Content

1. Experimental Study 1
2. NMR Data of the Adducts 1
3. NMR Spectral Copies of the Adducts 4

1. Experimental Study

Table 1. Solubility of CO_2 in Different Solvents.

Entry	Solvent	Pressure/MPa	Temp./°C	Solvent Volume /mL	Solubility mg/mL
1	hexane	1.0	18	10	6.63
2	toluene	1.0	18	10	14.58
3	DMC	1.0	18	10	19.92
4	DMF	1.0	18	10	58.02

2. NMR Data of the Adducts

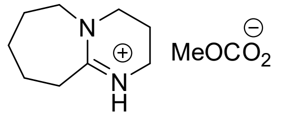

Colorless solid, ^1H NMR (400 MHz, DMSO-d_6): δ 8.98 (br, 1H, -N*H*), 3.48-3.39 (4H), 3.24-3.15 (5H), 2.68-2.65 (2H), 1.87-1.81 (2H), 1.64-1.57 (6H) ppm. ^{13}C NMR (100.6 MHz, DMSO-d_6): δ 164.5, 156.8, 52.9, 51.3, 47.8, 38.5, 31.7, 28.5, 26.5, 24.0, 19.6 ppm.

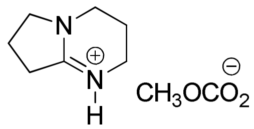

Colorless solid, ^{1}H NMR (400 MHz, DMSO-d_6): δ 10.15 (br, 1H, -N*H*), 3.53 (t, *J* = 7.0 Hz, 2H), 3.34 (t, *J* = 6.0 Hz, 2H), 3.27-3.14 (5H), 2.74 (t, *J* = 8.0 Hz, 2H), 2.02-1.94 (m, 2H), 1.88-1.82 (m, 2H) ppm. ^{13}C NMR (100.6 MHz, DMSO-d_6): δ 163.2, 157.0, 52.5, 51.3, 42.0, 38.4, 29.8, 18.7, 18.6 ppm.

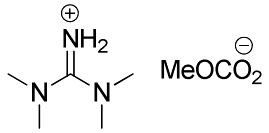

Colorless solid, ^{1}H NMR (400 MHz, DMSO-d_6): δ 7.87 (br, 2H, -NH_2), 3.22 (s, 3H), 2.83 (s, 12H) ppm. ^{13}C NMR (100.6 MHz, DMSO-d_6): δ 162.3, 156.5, 51.2, 39.3 ppm.

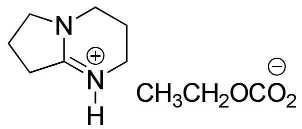

Colorless solid, ^{1}H NMR (400 MHz, DMSO-d_6): δ 10.06 (br, 1H, -N*H*), 3.69-3.64 (m, 1H), 3.54-3.51 (2H), 3.45-3.40 (1H), 3.34-3.32 (2H), 3.27-3.24 (2H), 2.75-2.71 (2H), 2.01-1.94 (m, 2H), 1.87-1.81 (m, 2H), 1.02 (t, *J* = 7.2 Hz, 3H) ppm. ^{13}C NMR (100.6 MHz, DMSO-d_6): δ 163.1, 156.6, 58.7, 52.4, 42.0, 38.5, 29.8, 18.8, 18.6, 15.5 ppm.

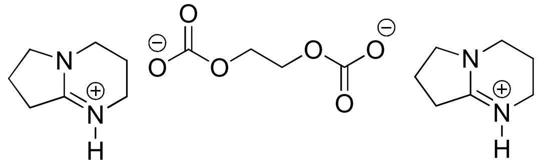

Colorless solid, ^{1}H NMR (400 MHz, DMSO-d_6): δ 9.24 (br, 1H, -N*H*), 8.51 (1H, -N*H*), 3.70 (2H), 3.45-3.37 (6H), 3.29-3.22 (8H), 2.62-2.58 (4H), 1.97-1.92 (m, 4H), 1.82-1.76 (m, 4H) ppm. ^{13}C NMR (100.6 MHz, DMSO-d_6): δ 162.1 (162.3), 156.7 (156.5), 65.6, 63.3 (63.0), 61.0 (60.8), 51.9, 42.2 (42.1), 39.8, 30.0 (29.9), 19.3 (19.2), 18.8 ppm.

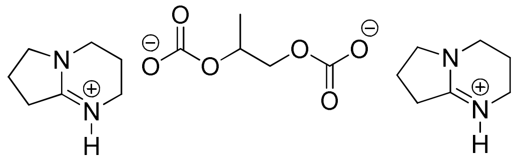

Colorless solid, ^{1}H NMR (400 MHz, DMSO-d_6): δ 9.08-8.81 (br, 2H, -N*H*), 3.63-3.43 (7H), 3.30-3.22 (8H), 2.65-2.61 (4H), 1.98-1.91 (4H), 1.83-1.77 (4H), 0.98 (t, 3H) ppm. ^{13}C NMR (100.6 MHz, DMSO-d_6): δ 162.2, 156.7, 69.8, 67.7 (67.5, 67.3), 65.8, 52.0, 42.2, 30.0, 19.2 (19.1), 18.7 ppm.

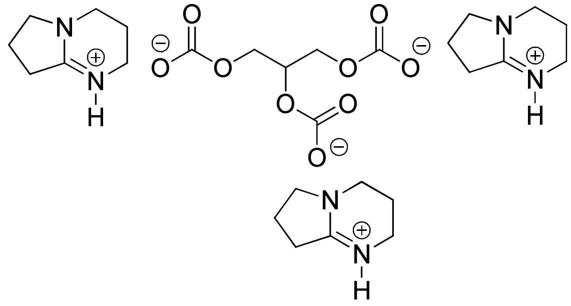

Colorless solid, ^{1}H NMR (400 MHz, DMSO-d_6): δ 8.60 (3H, -N*H*), 3.65-3.61 (2H), 3.42-3.39 (7H), 3.27-3.19 (13H), 2.55 (t, 6H), 1.95-1.88 (m, 6H), 1.79-1.74 (m, 6H) ppm. ^{13}C NMR (100.6 MHz, DMSO-d_6): δ 162.2, 157.5, 71.9, 66.7 (66.4), 63.7, 52.2, 42.6, 40.7, 30.5, 19.9, 19.3 ppm.

3. NMR Spectral Copies of the Adducts

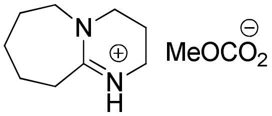

^{1}H NMR (400 MHz, DMSO-d_6) and ^{13}C NMR (100.6 MHz, DMSO-d_6)

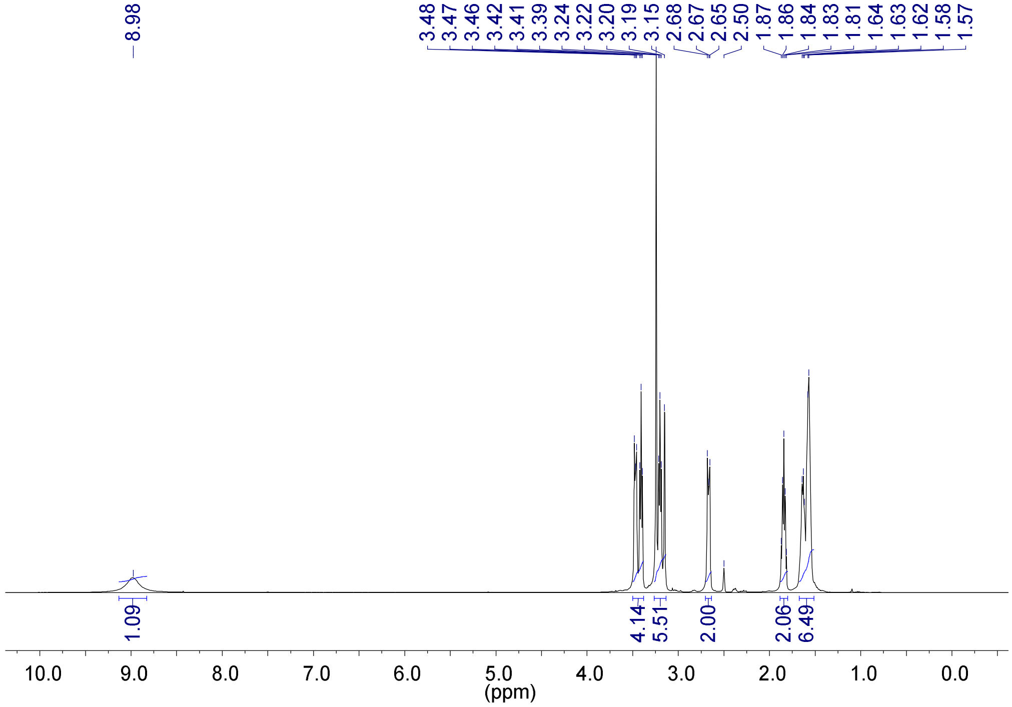

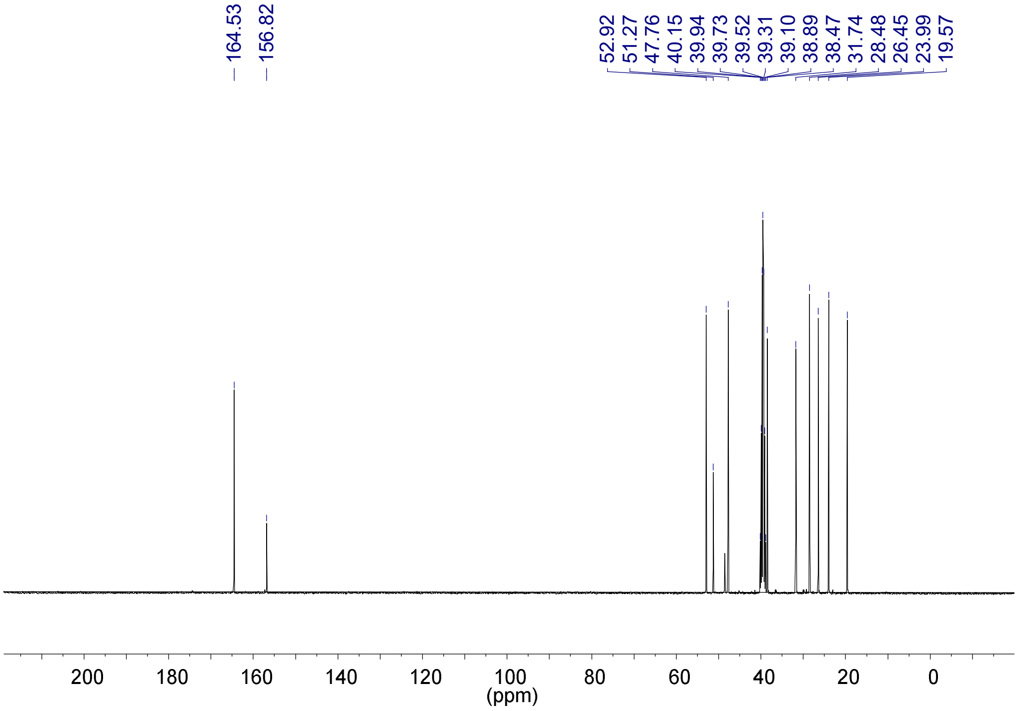

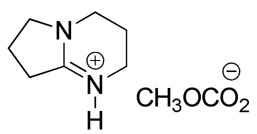

^{1}H NMR (400 MHz, DMSO-*d*$_6$) and ^{13}C NMR (100.6 MHz, DMSO-*d*$_6$)

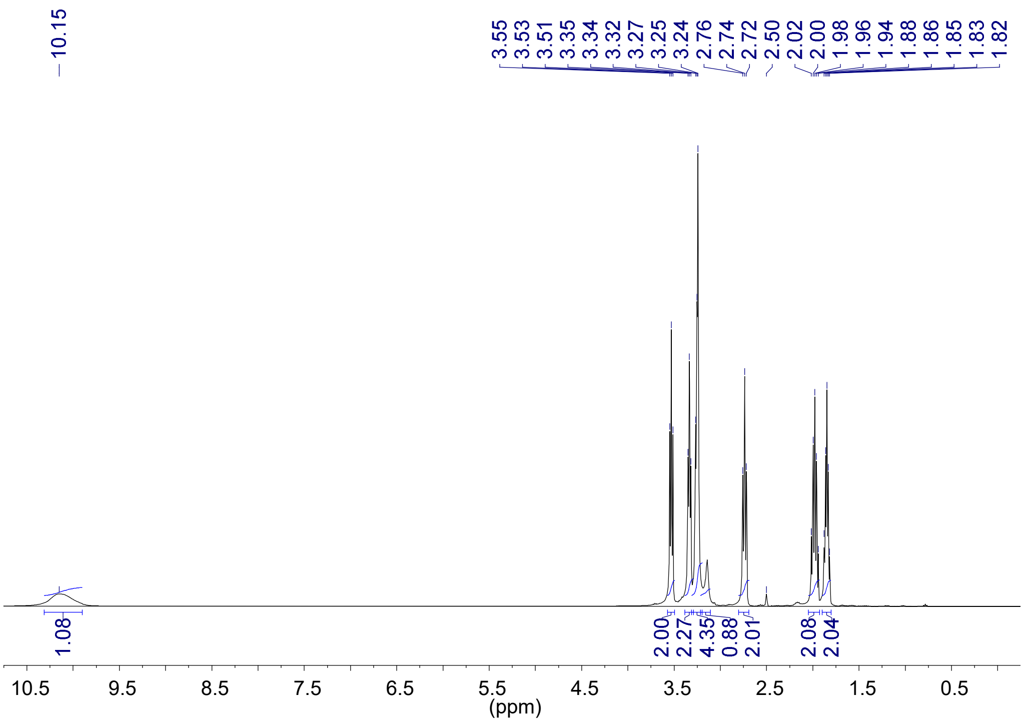

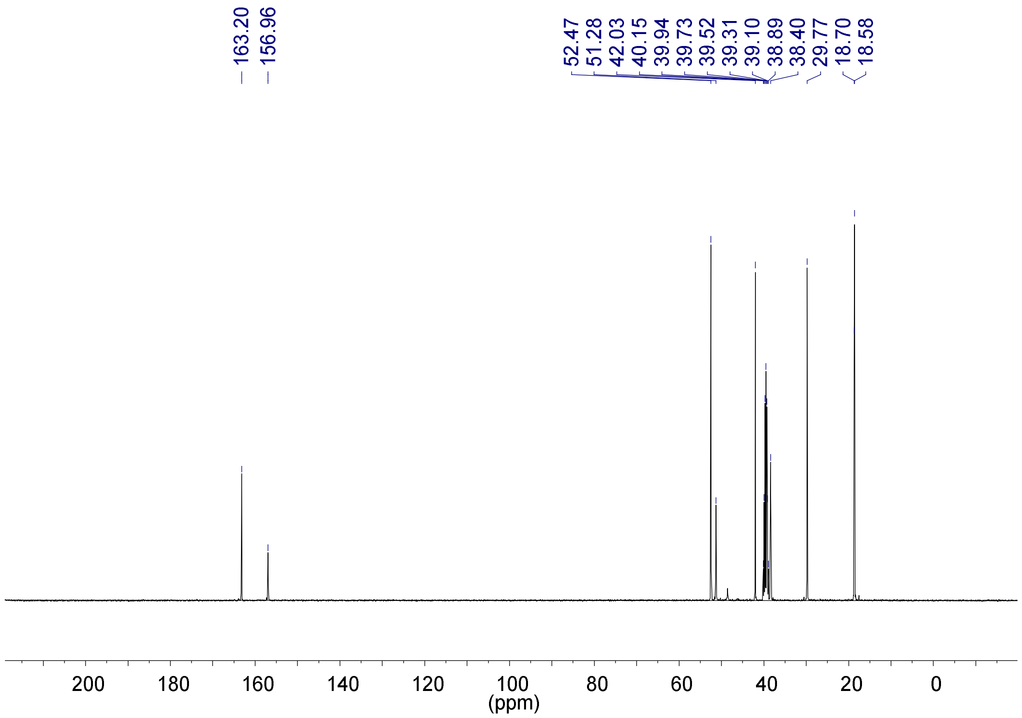

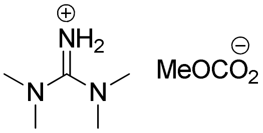

^{1}H NMR (400 MHz, DMSO-*d*6) and ^{13}C NMR (100.6 MHz, DMSO-*d*6)

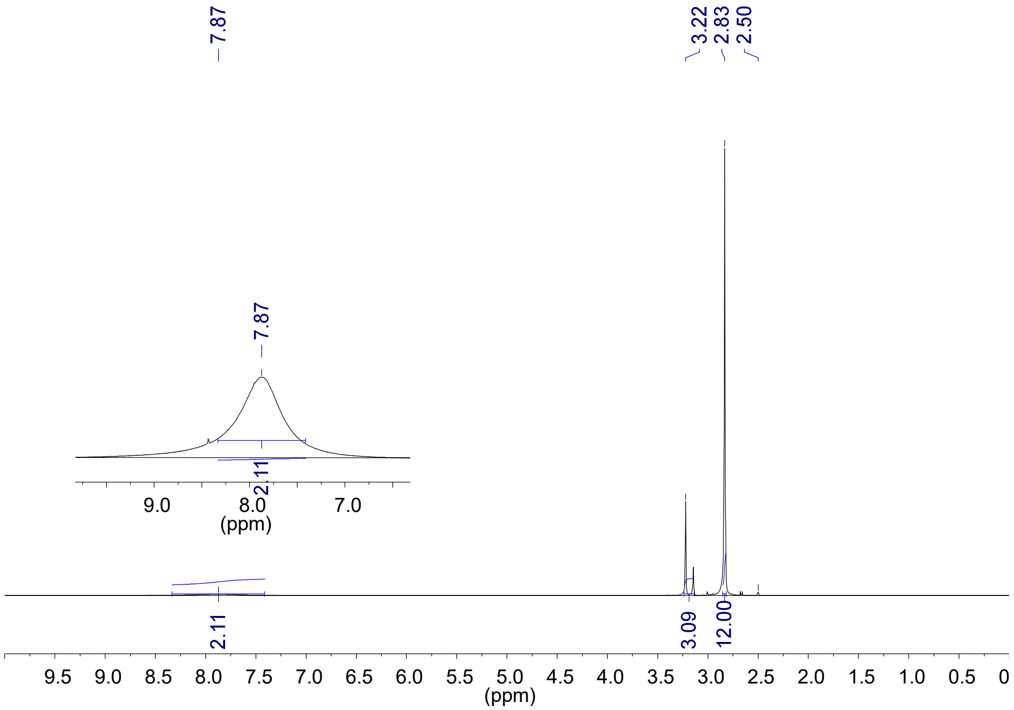

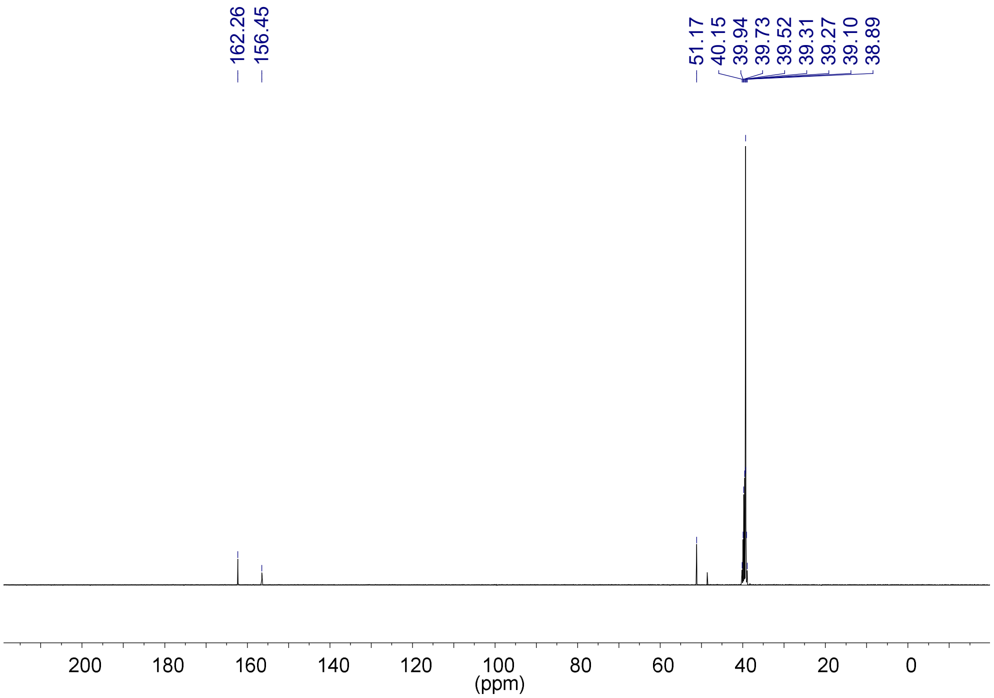

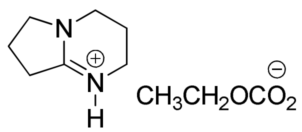

^{1}H NMR (400 MHz, DMSO-*d*6) and ^{13}C NMR (100.6 MHz, DMSO-*d*6)

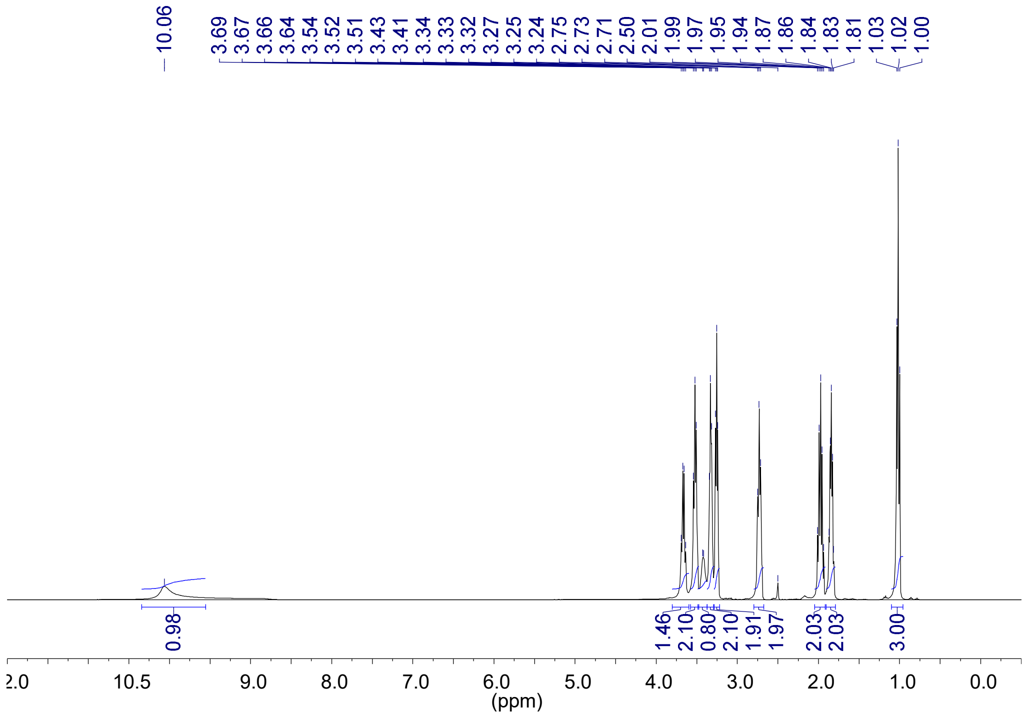

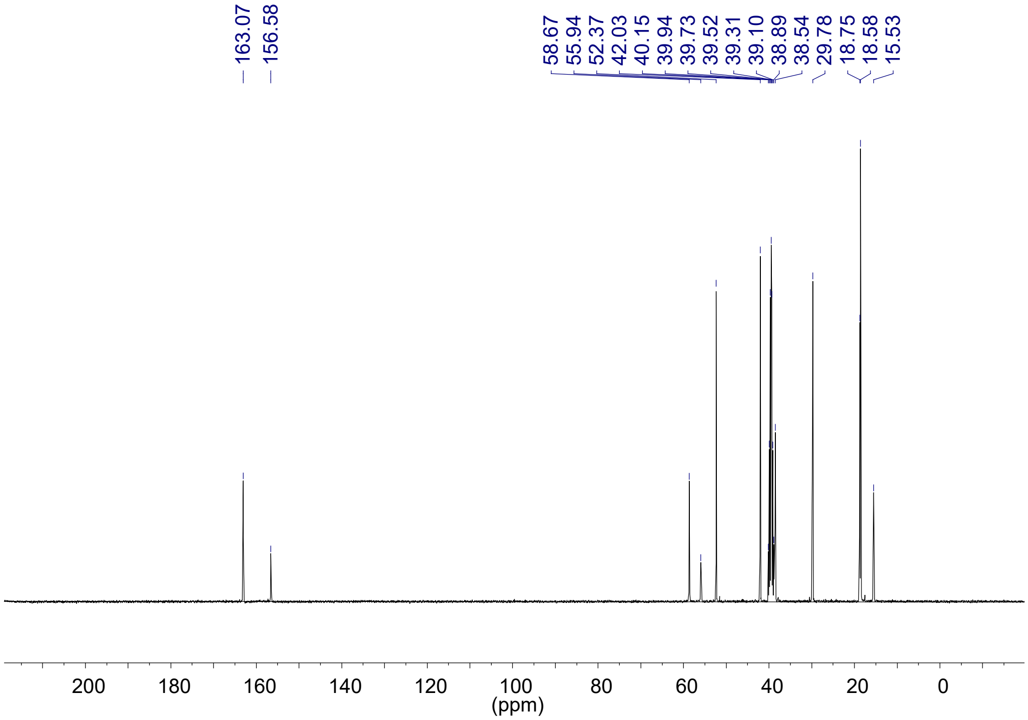

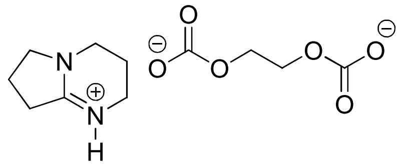

^{1}H NMR (400 MHz, DMSO-d_6) and ^{13}C NMR (100.6 MHz, DMSO-d_6)

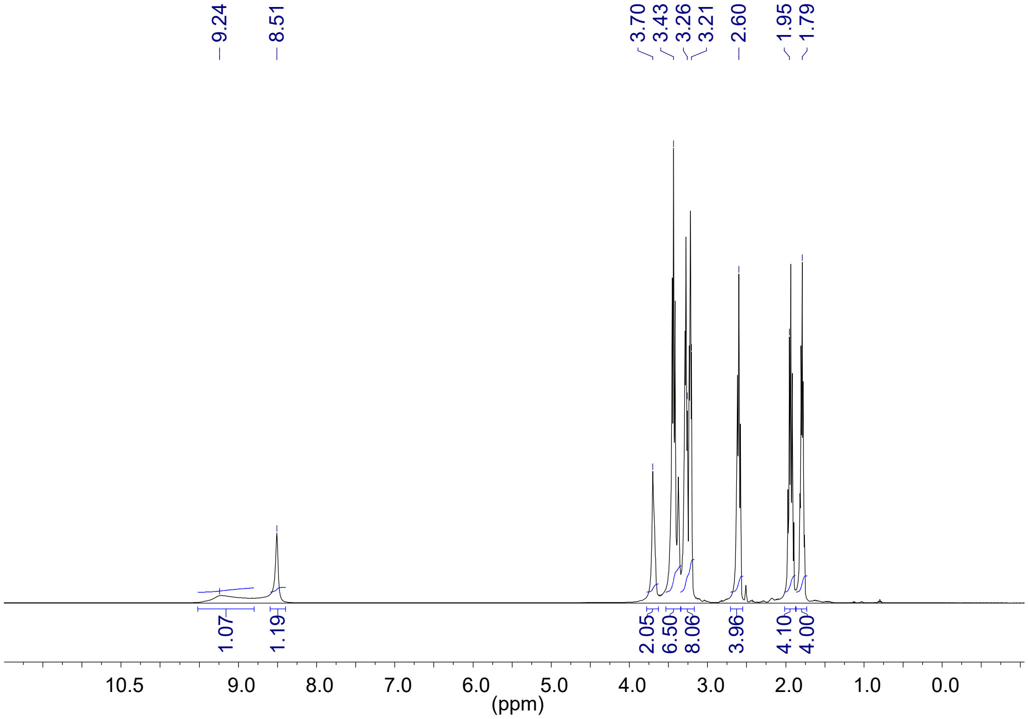

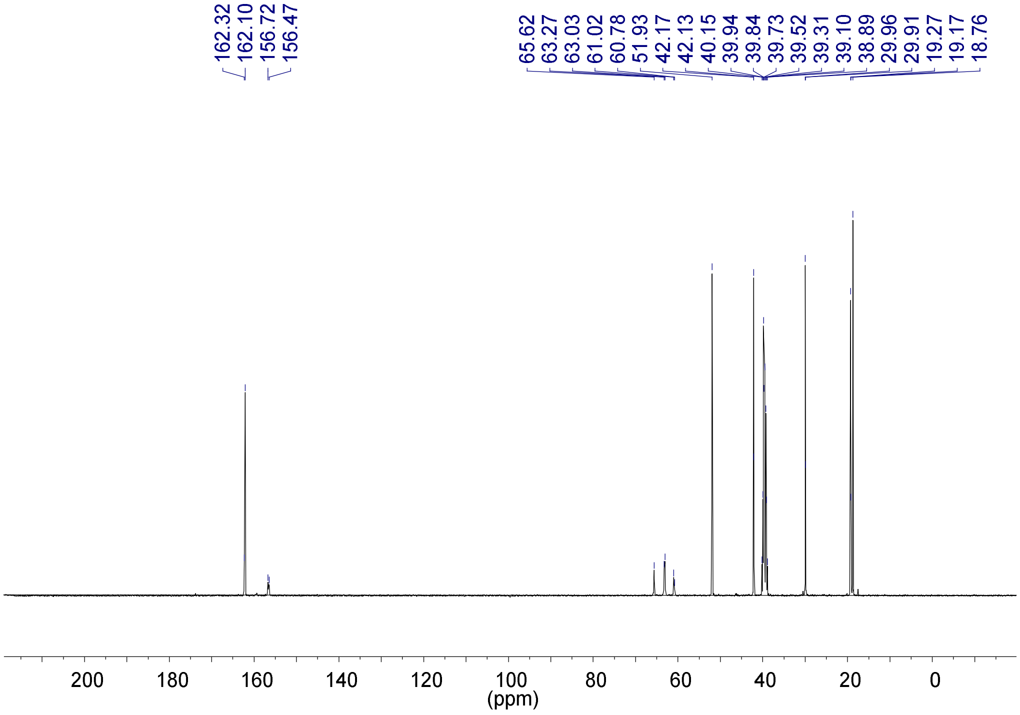

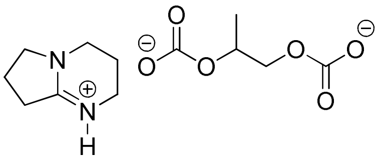

^{1}H NMR (400 MHz, DMSO-d_6) and ^{13}C NMR (100.6 MHz, DMSO-d_6)

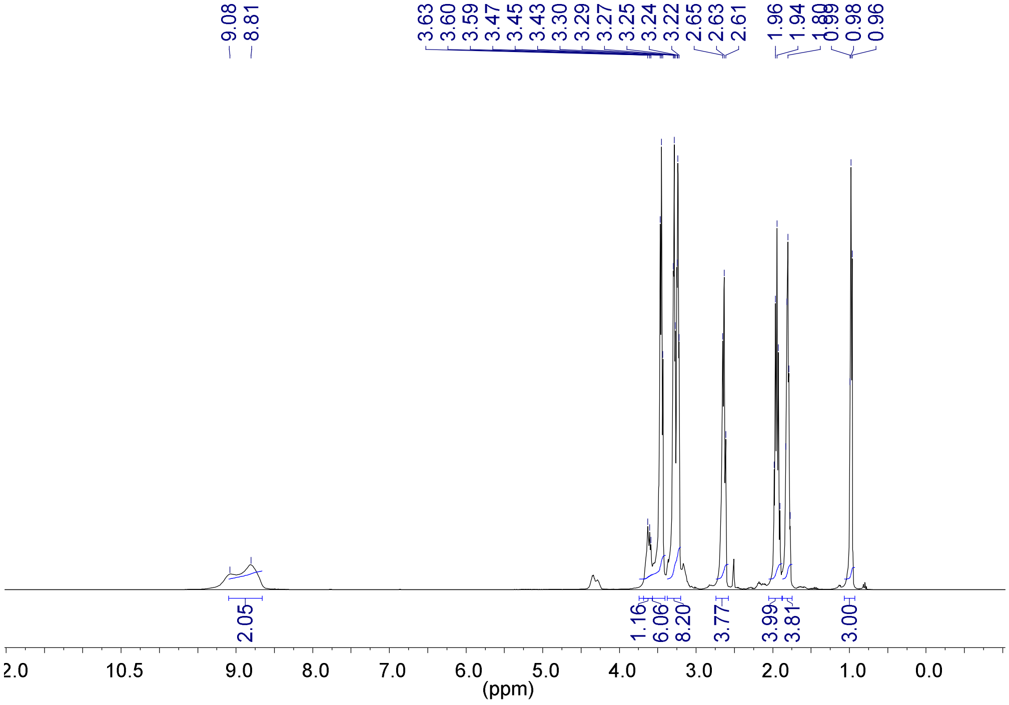

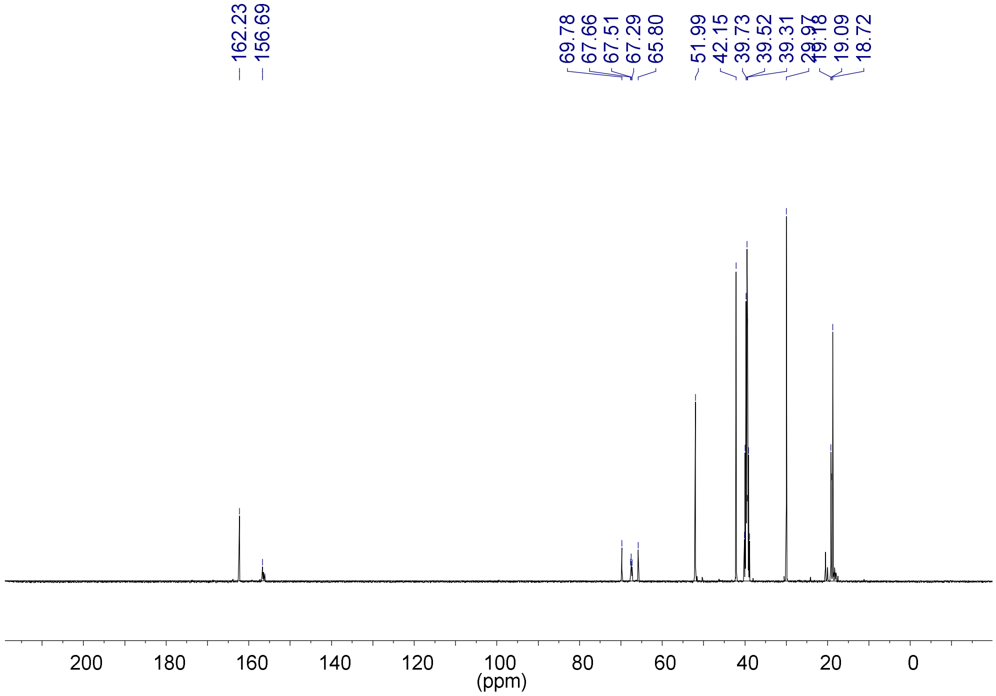

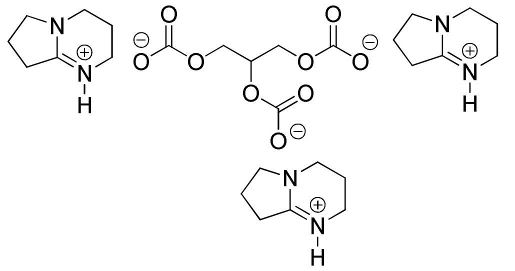

^{1}H NMR (400 MHz, DMSO-d_6) and ^{13}C NMR (100.6 MHz, DMSO-d_6)

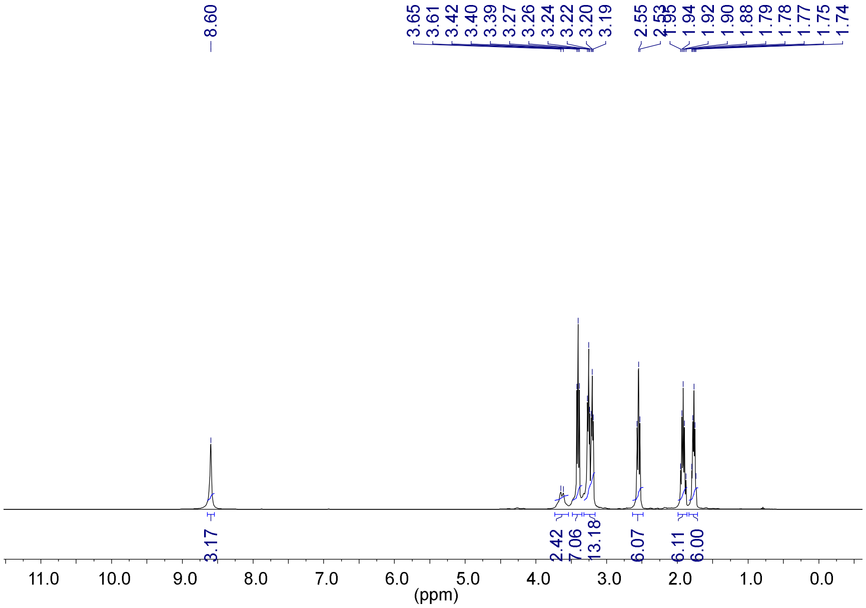

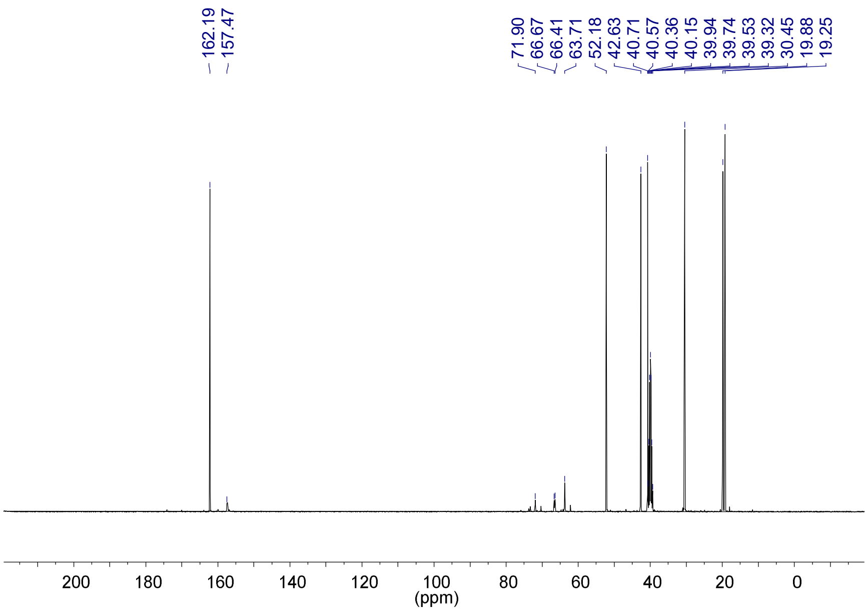

Supplement: Supplementary file 1 [file molecules-26-01735-s001.zip › molecules-1136974-supplementary.pdf]
